# Prisoners’ Perceived Violence and Hair Regulation in Hong Kong Prisons: Gender-Based Differences

**DOI:** 10.3389/fpsyg.2022.869898

**Published:** 2022-04-27

**Authors:** T. Wing Lo, Cora Y. T. Hui, Xin Guan, Sharon I. Kwok

**Affiliations:** ^1^Department of Social and Behavioural Sciences, City University of Hong Kong, Kowloon, Hong Kong SAR, China; ^2^School of Social Sciences, Western Sydney University, Penrith, NSW, Australia

**Keywords:** hair regulation, gender, violence, self-esteem, procedural fairness, negative emotion, prison, Hong Kong

## Abstract

Hair regulation is an essential policy for maintaining hygiene, security, and discipline in correctional institutions. However, the implementation of any hair-regulating policy should include a consideration of gender needs and differences. This study investigated Chinese prisoners’ perceived influence of hairstyles on their behavioral responses. Data were collected by means of a self-administered questionnaire survey from 500 male and 500 female prisoners in 11 correctional institutions of Hong Kong, China. Descriptive analyses and chi-square tests were used to explore the perceived violence of prisoners and gender differences. Mediation analysis was adopted to examine the prisoners’ perceived behavioral responses and mental and psychological well-being under different hairstyle situations, using self-esteem, procedural fairness, and negative emotional responses as mediators. The study found that male prisoners are inherently more tensive than the female group in terms of violent proclivities. In addition, perceived violent behavior is associated with hairstyle, and the influence path is gender related. Restrictive hair regulations that do not address unique social and cultural meanings and gender differences would decrease male prisoners’ self-esteem, while increasing all prisoners’ negative emotional responses and reducing their perceived procedural fairness. To maintain security inside institutions, we recommend short hair for male prisoners and long hair for female prisoners in Chinese prisons. Given that many prisons in Asian and African nations have an authoritarian style of governance similar to that of China, this study is of considerable international relevance.

## Introduction

The purpose of haircutting requirements in correctional institutions is to maintain prison hygiene, security, and discipline. As long hair can be used to aid attacking tactics against other inmates, and for committing suicide or concealing prohibited items, it could cause severe security concerns for prisons. In Hong Kong prisons, in order to maintain a secular, humane, and healthy prison environment, it is necessary to have such haircutting requirements listed in prison regulation “SO41-05” based on security issues, whereby male prisoners are required to cut their hair short, but the same is not the case for their female counterparts. However, it was held by a Hong Kong court (case HCAL 109/2014) that SO41-05 constituted sex discrimination, and the requirement of SO41-05 is therefore unlawful. There are no legitimate reasons to explain the inapplicability of the haircutting requirement in female prisons, as the security concern should also be applied to female prisoners. The court judgment has suggested that differences in treatment must only be justified when there is a legitimate, rational, and necessary aim to guarantee equality. If it is necessary to maintain prison security and to safeguard prisoners’ human rights, the same haircutting requirement should be applied to both male and female prisoners in a non-discriminatory way. That is, the issue is not about haircutting that violates human rights in correctional institutions but rather the selective requirement for male prisoners, but not female prisoners, to cut their hair short, which constitutes direct sex discrimination.

Against this backdrop, the present study was carried out with the objective to investigate the haircutting concerns from a perspective of gender differences. In particular, it assessed the security risks associated with different hairstyles of prisoners, with regard to the possibility of prisoners’ proclivity to violence and any other factors pertinent to these individuals’ mental and psychological well-being.

## Literature Review

### Cultural Meanings of Hairstyles and Gender Identity

Hair is a powerful agent to symbolize individual and group identity, gender, and sexuality, and a means of self-expression and communication (Ruberg, 2019). From a postmodernist perspective, hair symbolizes one’s self (Synnott, 1987; Fabry, 2016) as well as the borders of one’s body (Holton, 2020). Hair is regarded as “a tool through which discourses of power, control and authority are inscribed upon, encountered by and used against bodies in space” (Holton, 2020, p. 560). Hair is a key agent in producing and representing the body, specifically through the hairstyle that influences and transcends its margins (Holton, 2020). Hairstyles can influence judgments on the morality, sexuality, religiosity, and political persuasion of a person.

Although hair is a powerful symbol of individual identity with immense social significance, identity issues are universally different between men and women. As Stenn (2016) stated, universally and culturally women have longer hair than men. Extra time, wealth, and care are needed to keep long hair healthy. Consequently, long hair can be a status symbol (Stenn, 2016). Women’s long hair is also associated with femininity and facial attractiveness and is expected from women in regard to health status (Mesko and Bereczkei, 2004). By looking at perceptions with regard to different hair colors and lengths, Manning (2010) suggested that women who have short hair are believed to be less feminine than other women, as short hair is culturally assigned to men. In Chinese societies, long hair is also regarded as a sign of femininity and the physical attractiveness of young women (Zheng, 2016). From a social control perspective, long hair always means social regulation and obedience under religious or cultural conformity, while short hair in Chinese women could be signified as social freedom or defiance (Hiltebeitel and Miller, 1998; Zheng, 2016). Similarly, Weitz (2001) contended that short hair in United States women could be assigned a liberating meaning, signifying power and a feeling of beating the system.

On the other hand, short hair and shaven faces are symbolic of normative and morally upright males in the culture of mainstream Christianity (Singh, 1997). A good-mannered and cultural model of men would be with neat and tidy hair, and a clean and classic appearance (Hirschman and Brunswick, 2002). Hairstyles can also serve as a symbol of individual identity. Two studies by Leach (1958) and Hallpike (1969), anthropologists who studied the cultural meanings of men’s hair, argued that long hair is not merely a symbol of sexuality but also a symbol of being outside society and under less social control than other citizens. For example, it was found that long hair could mean independence and less recognizing of authority than short hair among male college students (Larsen and White, 1974). While gang violence (Melde and Esbensen, 2013) is always a concern in criminology and prison studies, Schneider (2004) contended that hairstyles were used by gangs in men’s prisons to maintain their group identity. He argued that short hair is the most universally recognizable hairstyle for gang identity and can also be an effective way to signify gang affiliation. These studies have shown that hairstyles do have cultural meanings in gender identity which are significantly different between men and women. Hairstyles have a unique role in gender perception to distinguish between men and women.

### Gender and Prison Violence

Prison violence has been associated with different factors (Sanhueza et al., 2021). One factor relates to prison management, such as poor staff–prisoner ratios, unfair treatment of prisoners, corruption, and deprived conditions inside prisons (Butler et al., 2021). Another factor relates to personal issues, such as prisoners’ psychological traits, mental health (Butler et al., 2021), offending records, and drug history (Celinska and Sung, 2014). Additionally, there are sociological aspects, such as prisoners’ responses to social discrimination (Bell, 2017). Finally, there are criminological aspects, such as gang-associated conflicts and fight for power and hierarchy in prison, as prison violence is more common among gang members incarcerated in men’s prisons (Fleisher and Decker, 2001; Gaes et al., 2002).

Gender as a variable in prison violence is not frequently studied in comparative research on men and women in custody. Hence, the results remain inconclusive. Some studies found that there is no significant effect of gender on prison violence (Bell and Lindekugel, 2015; Warren et al., 2018). Others suggested that violence is more common in men’s prisons compared to women’s prisons, and especially that instances of sexual violence (Wolff et al., 2007) and serious violence are much less common in women’s prisons (Craddock, 1996; Wulf-Ludden, 2013). Research has also revealed that men in prison are more prone to violence than are women (Sorensen and Cunningham, 2010; Wulf-Ludden, 2013). Other research, however, indicates that female prisoners can be as violent and conflict-laden as their counterparts in male correctional facilities, especially regarding low-level physical assaults (Wolff et al., 2007), and that women’s self-reported violence is significantly higher than figures recorded by institutions (Warren et al., 2018).

Prisons, like many other authoritarian institutions, illustrate gender relations that reflect the gender regime of a given institution. Although prisons are often single-gender institutions, they still are characterized by ideas of masculinity and femininity as in mixed-gender institutions. The gender relations encompass various practices that reflect definitions of masculinity and femininity, sexual divisions of labor, and other sexual ideologies that outline appropriate sexual behavior (Gorga, 2017). Studies on men’s prisons provide examples of gender hierarchies among prisoners: hyper-masculine men often are positioned at the top of the power hierarchy, while feminine (i.e., small and weak) men are often at the bottom (Donaldson, 2001; Hensley et al., 2003).

Women’s prisons also share a similar subculture as men’s prisons (Gorga, 2017). Women labeled as “studs” are those with masculine hairstyle and appearance, often presenting as men to exploit and control feminine prisoners for personal advantage, such as special service or financial support (Gorga, 2017). Prison staff also indirectly produce gender differences inside the institutions (Britton, 2003), causing unfairness to those female prisoners with more masculine traits, such that the studs are subjected to more surveillance and scrutiny by the staff, because they may cause more trouble and misconduct. Thus, the prisoners’ physical appearance of masculinity and femininity contributes to shaping gender hierarchy and possibly causes gender exploitation in women’s prisons.

When examining factors in relation to gender differences in prison violence, Thomson et al. (2019) suggest that affective psychopathic traits explain the violence of female prisoners, regardless of impulsivity. In addition, there are other predictive factors on female prisoners’ misconduct and violence, such as level of social support (Celinska and Sung, 2014). For male prisoners, masculinity or “doing-gender” (using violence to express gender identity), and using violence to compete for power and resources, are the dominant explanations of violence in men’s prisons (Michalski, 2017).

### Hair Regulation and Gender Concerns in Prisons

Restrictive hair regulations in prisons are mainly based on four justifications: hygiene, workplace safety, prisoners’ identification, and prison security. The first reason is to avoid skin and louse infection and to reduce the difficulties of identifying possible skin disorders. Maintaining prisoner cleanliness with short hair and shaving helps to minimize the costs of bathing and the plumbing maintenance costs that may be caused by clogs and backups. Second, for prisoners who may need to work in food service and with industrial machinery, short hair can ensure hygiene and safety. It can also reduce the chances of conflict between staff and prisoners when requiring them to wear a face guard or a hair net. Third, short hair and a shaved face can clarify facial characteristics for prisoner identification. Restrictive hair regulations can help administrators to quickly identify prisoners and prevent them from hiding facial characteristics. Finally, regulating long hair can reduce the chance of concealing contraband and thus minimize the additional efforts of prison staff to search for contraband. Long hair can also be used in fighting tactics when violence occurs (Singh, 1997); for example, long hair can prompt attackers to pull an opponent’s hair (Tjaden and Thoennes, 1998; Rippon, 2000).

As hair symbolizes one’s identity, forced haircutting is strongly connected to shame. For male prisoners, haircutting can have a special meaning that they have become separated from their previous life and people with whom they were associated prior to imprisonment. Indeed, their identities are changed to match previously admitted prisoners (Serico, 2015). Cutting hair, and especially shaving the heads of convicts, could signify their conviction and the subsequent life under rigid discipline and loss of freedom as a form of punishment (Hirschman and Brunswick, 2002).

Historically, hair shearing has unique meaning for women. It can be regarded as a form of shame and desexualization, intended to take away women’s dignity (Warring, 2006) and representing a symbolic castration. That is, their sexual identity is taken away, thus diminishing their power of physical attractiveness. Restrictive hair regulations exploit women’s agency in aesthetic choices, limiting their ability to “do gender” (engage in actions that are subject to evaluation by others as being appropriate for a woman) and to maintain and express one’s self in both public and private spheres (Holton, 2020). Mandatory hair regulations, especially cutting women’s hair without their consent and acceptance, can be seen as destroying the woman’s self.

From this perspective, cutting female prisoners’ hair on an involuntary basis can be regarded as disciplining the body and imposing “secondary punishment” to the convicted (Ruberg, 2019). Restrictive hair regulations in women’s prisons cause a continual affront to the female prisoners’ self-esteem. For the majority, hair signifies their dignity, and cutting their hair and prohibiting them from maintaining hair rituals are regarded as a more severe form of punishment than incarceration. When hair becomes a critical claim to self-esteem, the restriction and control over hair during incarceration can be regarded as a form of bodily violence against female prisoners (Labotka, 2014).

Another study on regulating women’s hair has supported this perspective. A study of restrictive haircuts among newly recruited female police officers revealed that the women regarded cutting their hair as the loss of agency, hence having an impact on their perceived self-image. They expressed an impact on their personal lives, including on relationships with significant others, as they no longer embody their former selves when they return to their private lives (Kringen and Novich, 2018).

To summarize, the literature suggests that hairstyles have unique social and cultural meanings for both men and women. Culturally women have long hair and men short. There are gender differences in prison violence. While male prisoners’ violence is mainly related to power and resources and the maintenance of a masculine identity, female prisoners tend to commit less serious violence, which is often caused by emotional and affective responses and the lack of social support. While there are legitimate reasons for correctional institutions to implement restrictive hair regulations, such policies may serve as another type of punishment or violence against the prisoners.

## Research Methodology

### Objectives

There is no preexisting research that has investigated how hairstyles impact violence in Chinese prisons, including its gender differences and mediating factors. Against this backdrop, the present study aimed to investigate haircutting concerns in terms of Chinese prisoners’ perceptions of violence. In particular, it assessed the security risks associated with different hairstyles of male and female prisoners in Chinese prisons, in regard to their proclivity to violence and any other factors pertinent to their social, mental, and psychological well-being. These factors include the prisoners’ sense of procedural fairness, negative emotional consequences, and self-esteem.

### Participants and Data-Collection Procedures

After ethical approval was sought from the institution, a pilot study was conducted. Four focus groups were held with 20 male and 20 female correctional officers to solicit opinions on the daily operations, security control, and violence risks in correctional institutions. Moreover, we interviewed three male and two female ex-prisoners identified through our own research network to collect the same kind of information. The pilot study aimed to collect firsthand material to facilitate the construction of a survey questionnaire for the main study. Each focus group and interview lasted about 80 min, and the data were transcribed by research assistants. The transcription was validated by a researcher who conducted the group or interview. After the transcription was confirmed, thematic analysis was conducted through the following steps: (1) familiarizing with the data, (2) generating initial codes, (3) searching for themes, (4) reviewing themes, and (5) defining and naming themes (Braun and Clarke, 2006). Eventually, we identified the following themes for the construction of the questionnaire: (1) aggression, (2) individual violence, (3) negative emotional responses, (4) procedural fairness, (5) self-esteem, and (6) the use of weapons in prison violence.

The main study was conducted between December 2017 and January 2018, using purposive sampling. With the help of the Correctional Services Department of Hong Kong, we sent out invitations to recruit 500 male prisoners and 500 female prisoners who were imprisoned in 11 correctional institutions. The number of participants recruited was proportional to the population size of the correctional institutions. The breakdown of the number of participants from each institution is shown in Table 1. Participants participated in the study on a voluntary basis without any incentive after giving informed consent. They were released from their daily work routines and organized into small groups in a comfortable room. With the assistance of prison staff, a research assistant randomly distributed one of the two versions (long or short hair) of the questionnaire and a consent form to each participant. They read and signed the consent form, which explained the study’s confidentiality and anonymity, before they proceeded to answer the questions on their own. For those who had queries on the questions, the research assistant provided guidance. Participants were also assured that they were free to withdraw from the study at any time. The following measures were adopted to ensure the anonymity and confidentiality of the participants’ responses: the questionnaires were anonymous, that is, respondents were not required to fill in their names, and there were no items that could identify them; the questionnaires were self-administered, without any discussion with other participants or staff; and upon completion, the questionnaires were put into envelopes provided by the research team and collected by the research assistant.

**TABLE 1:** Number of participants.

	Male	Female
● Male prison 1	162	/
● Male prison 2	86	/
● Male prison 3	52	/
● Male prison 4	52	/
● Male prison 5	46	/
● Male prison 6	36	/
● Male prison 7	36	/
● Male prison 8	30	/
● Female prison 1	/	356
● Female prison 2	/	102
● Female prison 3	/	42
Total	500	500

### Materials and Measurements

**FIGURE 1:**
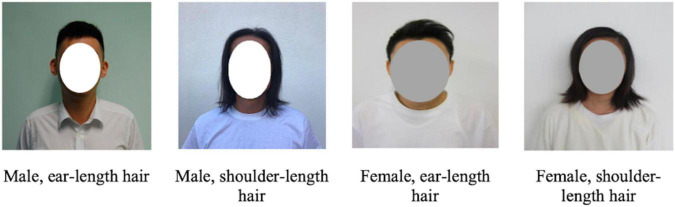
Hair style used in long hair vs. short hair scenarios.

There were two different versions of the questionnaire. All questions and statements were identical in both versions except for the scenario section. The scenario section in version A asked participants to imagine themselves retaining ear-length hair (hereafter “short” for simplicity, see Figure 1 for the hairstyles) during their custodial sentence and version B asked participants to imagine themselves retaining shoulder-length hair (hereafter “long” for simplicity) during their custodial sentence.

Moreover, the study adopted the following scales and items to assess aggression, observations of prison violence, self-esteem, procedural fairness, negative emotional responses and violent expressive behavior.

#### Aggression

We adapted the Chinese version of the Reactive-Proactive Aggression Questionnaire (RPAQ; Fung et al., 2009) to assess participants’ reactive and proactive aggression. Proactive aggression is described as “purposeful behavior aimed at gaining a reward of social dominance over others,” whereas reactive aggression is a “response to provocation or a perceived threat” (Fung et al., 2009, p. 473). The original RPAQ contains 23 behavioral items to be self-reported on a three-point scale, with 0 = never, 1 = sometimes, and 2 = often. After the pilot study with correctional institution staff, we dropped two items (namely “made obscene phone calls for fun” and “vandalized something for fun”) and modified the wordings of the remaining 21 items to better suit the adult prison population. Examples of our modifications include modifying the original item “used force to obtain money or things from others” to “used force to obtain cigarettes or drinks from others.” In our modified Aggression scale, 11 items assess reactive aggression (e.g., got angry when others threatened you) and 10 items assess proactive regression (e.g., had fights with others to show who was on top). Points are summated to form scores of reactive regression (max score = 22), proactive regression (max score = 20), and overall total aggression (max score = 42). The Cronbach’s alphas for proactive regression, reactive regression and total aggression are 0.96, 0.94 and 0.97, respectively.

#### Observations of Prison Violence

These questions were self-constructed by the research team. We asked participants to indicate their observations of prison violence and the use of weapons inside correctional institutions. An example of the questions and statements is “From what you see, prisoners frequently use the following tools/weapons in fights” (see Table 3). Questions on prisoners’ observation of common scenarios were constructed to reflect the situations of conflicts, confrontations, revenge seeking and use of violence to deal with conflicts inside correctional institutions (see Table 4).

#### Self-Esteem

We aimed to assess the effects of long and short hair lengths on a prisoner’s self-esteem. After informing participants, via the questionnaire, that they would be required to retain a certain hair length during their custodial sentence (long or short, depending on questionnaire version), we asked them to rate their agreement on nine self-esteem items. To assess participants’ level of self-esteem, we translated the Rosenberg Self-Esteem Scale (Rosenberg, 1965) into Chinese and then back-translated it to confirm its original meaning. One item (“I wish I could have more respect for myself”) was dropped because of low consistency with the other items. The nine items we included in the present study are “I feel that I have a number of good qualities,” “I take a positive attitude toward myself,” “I feel that I am no good at all,” “I feel that I am a person of worth, at least on an equal plane with others,” “I would be inclined to feel that I am a failure,” “I feel that I am able to do things as well as most other people,” “I feel that I do not have much to be proud of,” “I feel useless” and “I am satisfied with myself.” Participants rated these items using a four-point scale ranging from 1 (strongly disagree) to 4 (strongly agree). The Cronbach’s alpha for the self-esteem scale is 0.88.

#### Procedural Fairness

We wanted to assess the effects of long and short hair lengths on a prisoner’s perception of the procedural fairness of correctional institutions (CI). We translated and adapted the procedural fairness scale from Reisig and Mesko (2009). We dropped the item “The guards are courteous to inmates” because the emphasis of commands in CIs is often at odds with courtesy. The five items we used are “CI staff treat prisoners with respect,” “CI staff treat prisoners fairly,” “CI staff explain their decisions to prisoners,” “CI staff make decisions based on facts and not personal opinions” and “CI staff take the time to listen to prisoners.” Participants were asked if the hair length requirement would be enforced, what is their perception toward CI staff maintaining procedural fairness. Participants rated the survey items using a four-point scale ranging from 1 (strongly disagree) to 4 (strongly agree). The Cronbach’s alpha of the procedural fairness scale is 0.95.

#### Negative Emotional Responses

We assessed the extent to which long and short hair lengths arouse prisoner’s negative emotional responses. We constructed a six-item scale assessing participants’ negative emotional responses. Participants were asked if the hair length requirement would be enforced and their emotional response. They rated their feelings of being disrespected, inferiority, furiousness, bitterness, anxiety and sadness on a five-point scale, with 0 meaning a lack of such feeling and 4 meaning a strong feeling. The Cronbach’s alpha of the negative emotional responses scale is 0.98.

#### Violent Expressive Behavior (Outcome Variable)

Participants were asked to indicate the possibility of them harming themselves, throwing things, hitting others, pulling other’s hair and threatening/swearing at others on a three-point scale if their hair was required to be a certain length (long vs. short), with 0 = certainly not, 1 = maybe, and 2 = certainly (see Tables 7, 8). These five items were selected based on the information obtained from four focus groups with prison staff and five interviews with ex-prisoners. The Cronbach’s alpha of violent expressive behavior scale is 0.94.

### Analytical Procedures

Apart from gathering descriptive statistics, we also conducted chi-square tests to compare the significance in differences between the two genders, followed by Cramer’s V, which is an effect size measurement for the chi-square test of independence. It measures how strongly two categorical fields are associated. Cramer’s V refers to the magnitude of effect size (ES): ES ≤ 0.2 refers to fields that are only weakly associated; 0.2 < ES ≤ 0.6 to the fields that are moderately associated; and ES > 0.6 to the fields that are strongly associated. In addition, multivariate analysis was used because we have assumed that a variety of factors jointly affect prisoners’ tendency to engage in violent expressive behavior. We used hierarchical multiple regression to assess how (i.e., positively or negatively) and to what extent certain predictors (e.g., hair length, aggression tendency) jointly affect the outcome (i.e., one’s tendency to engage in violent behavior). We used separate multiple regressions for male prisoners and female prisoners to assess the impact of various predictors on the likelihood that a participant would engage in violent expressive behavior. To further explore the pathways through which hair length would influence prisoners’ behavior, we set up three hypotheses and three mediators identified through the regression: self-esteem, procedural fairness, and negative emotional responses. To test the hypotheses, we conducted separate mediation analyses for male prisoners and female prisoners to assess the impact of various mediators on violent expressive behavior.

## Results

### Demographics

#### Male Prisoners

There were 500 male participants (Table 1). Most of them were aged between 31 and 40 (33.4%) and 21 and 30 (27%). Regarding their highest education level, 15.3% of them had attained primary school level or below. Most (41.7%) had attained a middle school level, and 29.2% a high school level (Table 2). Approximately 44% of them were single, and 32.3% married with children. The majority (80.6%) indicated that they were nationals/residents of China, Hong Kong, Taiwan or Macau, thus with Chinese cultural background, indicating that about one-fifth of them (19.4%) came from other countries. Besides these findings, 35.3% of them described themselves as religious. The majority were sentenced for drug-related offenses, accounting for 53.8%, followed by theft at 14.2%. Over half of them were jailed for not more than 4 years.

**TABLE 2:** Demographics.

	Male (*N* = 500)	Female (*N* = 500)
**Age**	%	%
21–30	27.0	25.0
31–40	33.4	38.0
41–50	22.4	29.6
51–60	12.8	5.7
61 or above	4.2	1.6
**Highest level of education obtained**		
Primary school or below (grade 6 or below)	15.3	21.4
Middle school (grades 7 to 9)	41.7	41.1
High school (grades 10 to 11)	29.2	25.9
Matriculation (grades 12 to 13)	4.4	3.5
Post-secondary certificate or community college	3.0	4.5
Bachelor degree or above	4.6	2.9
N.A./others	1.6	0.8
**Marital status**		
Married with children	32.3	38.7
Married with no children	10.0	17.4
In a relationship	10.7	13.8
Single	44.1	25.8
N.A./others	3.0	4.3
**Nationality/permanent residency**		
China/Taiwan/Macau/Hong Kong	80.6	78.4
Others	19.4	21.6
**Religion**		
No, I am not religious	64.7	65.4
Yes, I am religious	35.3	34.6
**Principal offenses charged**		
Possession/trafficking/manufacturing of drugs	53.8	27.4
Theft	14.2	16.8
Deception	6.6	9.4
Robbery	3.8	0.6
Wounding/assault	3.8	0.8
Rape or indecent assault	1.6	0
Blackmail	0.8	0
Murder	1.6	0
Burglary	3.8	1.2
Domestic violence	0	0.2
Gang-related offenses	1.4	0
Forgery	2.6	14.8
Unlawfully remaining in Hong Kong	5.0	20.8
Prostitution	0.4	1.8
Others	7.2	7.2
**Length of sentence (month)**		
12 or below	11.6	55.2
13–24	17.8	28.6
25–36	15.3	4.6
37–48	11.6	2.6
49–60	7.4	1.4
61–72	5.9	1.2
73–84	4.4	0.9
85–96	4.4	0.9
97–108	2.2	0.3
109–120	3.2	0.6
121 or above	16.0	3.8

**TABLE 3:** Type of tools/weapons used in fights.

Type of tools/weapons used in fights	Gender				Pearson’s chi-square	Cramer’s V (ES)
	Male (*N* = 500)		Female (*N* = 500)			
	%	No. (adjusted residuals)	%	No. (adjusted residuals)		
● Sharpened toothbrush	17.4	87 (9.8)	0	0 (−9.8)	95.29 (*p* < 0.001)	0.309 (*p* < 0.001)
● Batteries	8.6	43 (6.3)	0.4	2 (−6.3)	39.12 (*p* < 0.001)	0.198 (*p* < 0.001)
● Cup/tumbler	33.8	169 (12.7)	2.8	14 (−12.7)	160.69 (*p* < 0.001)	0.401 (*p* < 0.001)
● Pen	30.2	151 (12.4)	1.6	8 (−12.4)	152.93 (*p* < 0.001)	0.391 (*p* < 0.001)
● Feces/urine	4.4	22 (3.1)	1.2	6 (−3.1)	9.41 (*p* < 0.01)	0.097 (*p* < 0.01)
● Saliva/spit	18.8	94 (5.6)	7.0	35 (−5.6)	30.98 (*p* < 0.001)	0.176 (*p* < 0.001)
● I did not see anything	34.2	171 (−16.3)	84.8	424 (16.3)	265.63 (*p* < 0.001)	0.515 (*p* < 0.001)

**TABLE 4:** Prisoners’ common observations inside the institutions.

	Male		Female		*t*-Test
	% *	Mean	% *	Mean	Sig.
**Situations facilitating the use of violence**					
● When fights/arguments occur, it is common for other prisoners to stand around and watch.	82.3	2.952	29.1	2.062	0.000
● Seeking revenge over previous conflicts is common.	78.5	2.926	13.4	1.864	0.000
● It is common for prisoners to incite others to create disturbance.	75.8	2.880	13.6	1.876	0.000
● The escalation of verbal confrontation to physical fights is common.	75.8	2.874	11.1	1.785	0.000
● Conflicts between prisoners are common.	74.8	2.891	22.3	1.964	0.000
● It is common for prisoners to settle outside disputes inside the institution.	59.7	2.761	5.0	2.510	0.003
● Storage of weapons is common among prisoners.	54.6	2.494	1.6	1.513	0.000
**Use of violence**					
● The use of violence to deal with conflicts/confrontations is commonplace.	71.2	2.813	8.1	1.772	0.000
● It is common for prisoners to use violence to take revenge on those who has bullied/hurt them before.	71.0	2.778	7.5	1.789	0.000
● It is common for prisoners to treat sex offenders violently.	62.5	2.852	5.4	2.397	0.000
● Fighting among prisoners (including the use of feces, urine or other liquids) is common.	61.9	2.615	2.6	1.557	0.000
● Gang fights (involving 5 or more prisoners) are common.	55.8	2.544	4.6	1.544	0.000
**Non-violent behavior**					
● It is common for prisoners to write letters to their families.	75.8	3.004	82.3	3.165	0.001
● It is common for prisoners to be concerned with social/political affairs.	60.8	2.639	57.8	2.636	0.954
● Reading is common among prisoners.	58.6	2.615	71.3	2.794	0.000
● Skincare or exercising is common among prisoners.	35.1	2.283	75.6	2.887	0.000
● Prisoners maintain a nice/cool hairstyle to enhance their confidence.	30.8	2.170	72.8	2.915	0.000
● Prisoners maintain a certain hair length to feel respected by others.	22.3	1.952	67.1	2.826	0.000

*% of participants who indicated “agree” or “strongly agree” to the statements on a four-point scale ranging from 1 = strongly disagree to 4 = strongly agree.

#### Female Prisoners

There were 500 female participants. Most of them were aged between 31 and 40 (38%) and 41 and 50 (29.6%). Regarding their highest education level, most of them had attained a middle school level, accounting for 41.1%. Less than one quarter of them (21.4%) completed primary school and another quarter (25.9%) completed high school. Regarding their marriage status, 25.8% of them were single, and 38.7% of them were married with children. The majority (78.4%) were nationals/residents of China, Hong Kong, Taiwan or Macau, and about one-fifth of them (21.6%) came from other countries. Besides these findings, 34.6% of them were religious. Quite a large number of the female prisoners were sentenced for drug-related offenses, accounting for 27.4%, while the two other main offenses were remaining in Hong Kong unlawfully, accounting for 20.8%, and theft at 16.8% (Table 2). Over half of them (55.2%) were jailed for 1 year or less and more than one quarter (28.6%) for 1 to 2 years.

### Use of Weapons in Correctional Institutions

We asked participants to report all types of tools or weapons they saw being used in fights. Table 3 illustrates the use of tools and weapons d curing fights inside correctional institutions. Only about one-third (34.2%) of male prisoners reported not seeing the use of weapons during fights. Cups or tumblers and pens were the most common types of weapon used, reported by 33.8% and 30.2% of male prisoners, respectively. Saliva and sharpened toothbrushes were also commonly used, reported by 18.8% and 17.4% of male prisoners, respectively. On the other hand, the use of weapons during fights does not seem to be common inside female institutions. Around 85% of female prisoners reported not seeing the use of weapons during fights. Table 3 shows the chi-square test result. All Pearson’s chi-squares are significant (*p* < 0.05), which means that there is a correlation between gender and the use of weapons. Cramer’s V shows that the use of cups/tumblers (ES = 0.401, *p* < 0.001), pens (ES = 0.391, *p* < 0.001) and sharpened toothbrushes (ES = 0.309, *p* < 0.001) is moderately correlated with gender. Moreover, all adjusted residuals are higher than three, which indicates that male prisoners tend to use all types of weapons in Table 3, while female prisoners do not.

### Observations of Violence Inside Correctional Institutions

We asked participants to rate their agreement with statements regarding their observations inside the correctional institutions on a four-point scale ranging from 1 = strongly disagree to 4 = strongly agree. Table 4 shows that about three quarters (74.8%) of male prisoners but less than one quarter (22.3%) of female prisoners indicated that conflicts between prisoners are common, as was fighting among male prisoners (61.9% versus 2.6% of female prisoners, *p* < 0.001). In addition, about 71% of male prisoners indicated that violence was commonly used to deal with conflicts and seek revenge inside institutions against only 8.1% and 7.5% of female prisoners (*p* < 0.001), respectively. More than half (55.8%) of male prisoners also indicated that gang fights involving five or more people are common inside institutions against only 4.6% of female prisoners (*p* < 0.001). More than half of the male prisoners (54.6%) reported that the storage of weapons is common among them as compared to only 1.6% of female prisoners (*p* < 0.001).

Concerning non-violent behavior, it is more common for female prisoners to write letters to families, read, exercise and use skincare than male prisoners (*p* < 0.001) (see Table 4). It is important to note that female prisoners commonly agreed that the maintenance of a certain hair length or hairstyle is needed to maintain or enhance one’s self-esteem. About 67% of female prisoners indicated that prisoners maintain a certain hair length to feel respected by others against 22.3% of male prisoners, and 72.8% indicated that prisoners maintain a nice/cool hairstyle to enhance their confidence against 30.8% of male prisoners. The findings suggest that a presentable hairstyle and hair length are linked to female prisoners’ self-esteem.

Tables 3–5 show that weapon usage and violence are common inside male institutions, and even male prisoners themselves expressed concerns that allowing male prisoners to retain long hair could exacerbate the security risks. We asked participants to indicate their concerns over allowing prisoners to retain long hair, and only 19.4% of male prisoners did not express any worry (Table 5). Because of the high frequency of storing weapons and using them inside male institutions, 44.6% of male prisoners reported being concerned that other prisoners could store weapons inside their hair if they were allowed to retain long hair. More than one-third (36.6%) of male prisoners were concerned that prisoners with long hair have higher risks of being attacked as others could pull their hair more easily. The overwhelming majority (86.8%) of female prisoners were not concerned that allowing prisoners to retain long hair would exacerbate the security risks. Very few female prisoners (2.2%) expressed the concern that long-haired prisoners could hide weapons in their hair. This is not surprising as we see in the tables, as weapon storage and the use of violence are not common inside female institutions.

**TABLE 5:** Concerns of allowing prisoners retain long hair.

Type of concerns of allowing prisoners retain long hair	Gender				Pearson’s chi-square	Cramer’s V

	Male (*N* = 500)		Female (*N* = 500)			
	%	No. (adjusted residuals)	%	No. (adjusted residuals)		
Prisoners could hide weapons inside their hair	44.6	223 (15.8)	2.2	11 (−15.8)	250.74 (*p* < 0.001)	0.501 (*p* < 0.001)
Bad hygiene inside the institution	45.4	227 (15.1)	4.2	21 (−15.1)	227.54 (*p* < 0.001)	0.477 (*p* < 0.001)
Prisoners with long hair have higher risks of being attacked	36.6	183 (12.7)	4.2	21 (−12.7)	161.62 (*p* < 0.001)	0.402 (*p* < 0.001)
Prisoners could use their long hair to hang themselves	17.4	87 (8.5)	1.6	8 (−8.5)	72.59 (*p* < 0.001)	0.269 (*p* < 0.001)
Prisoners could use their long hair as a weapon	12.6	64 (6.9)	1.4	7 (−6.9)	48.17 (*p* < 0.001)	0.219 (*p* < 0.001)
Hair could get caught in the machine while working	28.2	141 (10.1)	4.6	23 (−10.1)	101.56 (*p* < 0.001)	0.319 (*p* < 0.001)
I am not worried about the above situations	19.4	97 (−21.4)	86.8	434 (21.4)	456.03 (*p* < 0.001)	0.675 (*p* < 0.001)

In Table 5, all Pearson’s chi-squares are significant (*p* < 0.001), which means that there is a correlation between gender and concerns over allowing prisoners to retain long hair. Specifically, male prisoners more than female prisoners were worried about three main concerns: prisoners hiding weapons inside their hair (ES = 0.501, *p* < 0.001), bad hygiene inside the institution (ES = 0.477, *p* < 0.001) and being easily attacked because of long hair (ES = 0.402, *p* < 0.001). Additionally, the majority of adjusted residuals were higher than three. Indeed, male prisoners tend to consider all issues related to long hair in Table 5, while female prisoners tend to not.

### Individual Levels of Aggression

We found that male prisoners indicated higher levels of proactive (*M* = 6.41, SD = 5.9) and reactive aggression (*M* = 9.26, SD = 5.79) when compared with female prisoners (*p* < 0.001, see Table 6). Male prisoners reported that they were more likely to both purposely use aggression to gain rewards or social dominance and become aggressive as a response to provocation. On the other hand, female prisoners reported low levels of proactive (*M* = 0.73, SD = 1.83) and reactive aggression (*M* = 2.58, SD = 2.84).

**TABLE 6:** Means and standard deviations of aggression by prisoner groups.

	Male		Female		*t*-Test
Aggression *	*M*	*SD*	*M*	*SD*	Sig.
Proactive	6.41	5.90	0.73	1.83	0.000
Reactive	9.26	5.79	2.58	2.84	0.000
Total	15.67	11.20	3.31	4.17	0.000

*The maximum scores of proactive aggression, reactive regression, and total regression are 20, 22, and 42, respectively.

**TABLE 7:** % and chi-square test of male prisoner’s violent expressive behavior by hair length.

Type of violent expressive behavior		Male with short hair		Male with long hair		Pearson’s chi-square	Cramer’s V (ES)
		%	No. (adjusted residuals)	%	No. (adjusted residuals)		
Self-harm	Certainly not	87.5	217 (9.7)	46.4	116 (−9.7)	96.69 (*p* < 0.001)	0.441 (*p* < 0.001)
	Maybe	9.7	24 (−6.3)	32.8	82 (6.3)		
	Certainly will	2.8	7 (−6.2)	20.8	52 (6.2)		
Throw things	Certainly not	87.6	219 (11.2)	39.2	98 (−11.2)	120.01 (*p* < 0.001)	0.508 (*p* < 0.001)
	Maybe	11.2	28 (−8.0)	43.2	108 (8.0)		
	Certainly will	1.2	3 (−6.3)	17.6	44 (6.3)		
Hit others	Certainly not	85.6	214 (10.8)	39.0	97 (−10.8)	117.40 (*p* < 0.001)	0.485 (*p* < 0.001)
	Maybe	12.0	30 (−7.6)	42.2	105 (7.6)		
	Certainly will	2.4	6 (−6.0)	18.9	47 (6)		
Pull other’s hair	Certainly not	85.2	213 (10.3)	40.6	101 (−10.3)	110.94 (*p* < 0.001)	0.471 (*p* < 0.001)
	Maybe	13.2	33 (−7.0)	41.0	102 (7.0)		
	Certainly will	1.6	4 (−6.3)	18.5	46 (6.3)		
Swear at others	Certainly not	80.8	202 (9.9)	37.2	93 (−9.9)	101.22 (*p* < 0.001)	0.450 (*p* < 0.001)
	Maybe	16.0	40 (−6.6)	42.8	107 (6.6)		
	Certainly will	3.2	8 (−5.9)	20.0	50 (5.9)		

**TABLE 8:** % and chi-square test of female prisoner’s violent expressive behavior by hair length.

Type of violent expressive behavior		Female with short hair		Female with long hair		Pearson’s chi-square	Cramer’s V (ES)
		%	No. (adjusted residuals)	%	No. (adjusted residuals)		
Self-harm	Certainly not	63.5	158 (−5.5)	85.1	211 (5.5)	40.08 (*p* < 0.001)	0.284 (*p* < 0.001)
	Maybe	16.5	41 (1.5)	11.7	29 (−1.5)		
	Certainly will	20.1	50 (5.9)	3.2	8 (−5.9)		
Throw things	Certainly not	62.2	155 (−5.1)	82.7	206 (5.1)	29.61 (*p* < 0.001)	0.244 (*p* < 0.001)
	Maybe	17.7	44 (2.0)	11.2	28 (−2.0)		
	Certainly will	20.1	50 (4.7)	6.0	15 (−4.7)		
Hit others	Certainly not	74.4	183 (−2.5)	83.7	206 (2.5)	10.86 (*p* < 0.01)	0.149 (*p* < 0.01)
	Maybe	13.8	34 (0.5)	12.2	30 (−0.5)		
	Certainly will	11.8	29 (3.2)	4.1	10 (−3.2)		
Pull other’s hair	Certainly not	71.6	179 (−4.0)	86.2	212 (4.0)	18.72 (*p* < 0.001)	0.194 (*p* < 0.001)
	Maybe	15.6	39 (1.8)	10.2	25 (−1.8)		
	Certainly will	12.8	32 (3.7)	3.7	9 (−3.7)		
Swear at others	Certainly not	64.8	162 (−4.3)	81.9	203 (4.3)	19.85 (*p* < 0.001)	0.200 (*p* < 0.001)
	Maybe	20.4	51 (2.4)	12.5	31 (−2.4)		
	Certainly will	14.8	37 (3.4)	5.6	14 (−3.4)		

### Impact of Different Hair Lengths on Prisoners

We distributed two versions of the questionnaire to the prisoners, one on a short hair scenario and the other one long hair. Tables 7, 8 show that male and female prisoners reacted differently to long and short hair length requirements. Male prisoners had a lower tendency to engage in violent expressive behaviors when they thought they needed to retain short hair length. As displayed in Table 7, when male prisoners knew that they needed to retain a short hair length during their custodial sentences, only 1.2% to 3.2% reported they would “certainly” engage in violent expressive behavior, while 80.8% to 87.6% indicated they would “certainly not.” When male prisoners were informed they needed to retain long hair, 37.2% to 46.4% reported they would “certainly” engage in violent expressive behavior such as self-harm and hitting others. All Pearson’s chi-squares are significant (*p* < 0.001), which means that there is a correlation between retaining long hair and violent expression among male prisoners. Throwing things (ES = 0.508, *p* < 0.001), hitting others (ES = 0.485, *p* < 0.001) and pulling other’s hair (ES = 0.471, *p* < 0.001) are the three main violent expressions when male prisoners were asked to retain long hair. The results suggest that violent expressions are moderately associated with hair length.

On the other hand, female prisoners had a lower tendency to engage in violent expressive behavior when they thought they needed to retain long hair (see Table 8). When female prisoners were informed they needed to retain long hair, 81.9% to 86.2% reported they would “certainly not” engage in violent expressive behavior. Despite this, when female prisoners were informed they needed to retain a short hair length, 11.8% to 20.1% reported they would “certainly” engage in violent expressive behavior. In particular, 20.1% said they would harm themselves or throw things. All Pearson’s chi-squares are significant (*p* < 0.01), which means that there is a correlation between retaining short hair and violent expression among female prisoners. Self-harm (ES = 0.284, *p* < 0.001), throwing things (ES = 0.244, *p* < 0.001) and swearing at others (ES = 0.200, *p* < 0.001) were the three main violent expressions when female prisoners were asked to retain long hair; however, violent expressions were only weakly associated with hair length.

### Multivariate Analyses

#### Male Prisoners

Model 1 was statistically significant, *F*(5,363) = 45.122, *p* < 0.001, *R*^2^ = 0.375. Hair length (i.e., long hair vs. short hair) and aggression had significant partial effects on one’s tendency to engage in violent expressive behavior. In particular, requiring male prisoners to retain long hair (*B* = 0.588, SE = 0.051, β = 0.477, *p* < 0.001) had the largest effect on the outcome variable. Prisoners with higher levels of aggression (*B* = 0.426, SE = 0.074, β = 0.369, *p* < 0.001) were also more likely to engage in violent expressive behavior (Table 9). The self-esteem scale, procedural fairness scale, and negative emotional responses were entered in Model 2. Model 2 was statistically significant, *F*(3,360) = 56.434, *p* < 0.001, *R*^2^ = 0.571, meaning the relationship between the predictors and the outcome is unlikely to be caused by random chance. Hair length and aggression scores continued to exert significant partial, albeit reduced, effects on the outcome variable. Among the newly entered variables, self-esteem and negative emotional responses exerted significant effects. Those who reported a lower level of self-esteem (*B* = −0.173, SE = 0.056, β = −0.154, *p* = 0.002) and those who reported more negative emotional responses (*B* = 0.214, SE = 0.025, β = 0.464, *p* < 0.001) were more likely to engage in violent expressive behavior.

**TABLE 9:** Hierarchical multiple regression predicting tendency of violent expressive behavior for male prisoners.

Predictors	Model 1		Model 2	
	*B* (SE)	β	*B* (SE)	β
Constant	−0.697*** (0.087)		0.324 (0.245)	
Hair length (long vs. short hair)	0.588*** (0.051)	0.477	0.143* (0.058)	0.116
Frequency of instances of indiscipline	−0.015 (0.031)	−0.031	0.010 (0.026)	0.020
No. of violence-related crimes committed	0.016 (0.020)	0.033	0.006 (0.017)	0.012
Sentence length	0.000 (0.000)	0.028	0.000 (0.000)	0.001
Aggression (reactive and proactive)	0.426*** (0.074)	0.369	0.223** (0.064)	0.193
Self-esteem			−0.173** (0.056)	–0.154
Procedural fairness			−0.026 (0.042)	–0.032
Negative emotional responses			0.214*** (0.025)	0.464
*R* ^2^	0.383		0.581	
Adjust *R*^2^	0.375		0.571	
*R*^2^ change	0.383		0.197	
*F* for change in *R*^2^	45.122***		56.434***	

B refers to the unstandardized coefficient, β refers to the standardized coefficient, and SE refers to standard error. ***p < 0.001, **p < 0.01, *p < 0.05.

#### Female Prisoners

Model 1 was statistically significant, *F*(5,317) = 5.034, *p* < 0.001, *R*^2^ = 0.059. Hair length (i.e., long hair vs. short hair) and the frequency of committing acts of rebellion against prison discipline had significant partial effects on one’s tendency to engage in violent expressive behavior. In particular, requiring female prisoners to retain short hair (*B* = −0.261, SE = 0.065, β = −0.220, *p* < 0.001) had the largest effect on the outcome variable (Table 10). The self-esteem scale, procedural fairness scale, and negative emotional responses were entered in Model 2. Model 2 was statistically significant, *F*(3,314) = 26.249, *p* < 0.001, *R*^2^ = 0.240. The frequency of committing acts of rebellion against prison discipline continued to exert significant partial effects on the outcome variable. Among the newly entered variables, the perception of procedural fairness and negative emotional responses exerted significant effects, with the former emerging as the largest predictor. Those who reported a lower level of perceived procedural fairness by correctional institution staff (*B* = −0.326, SE = 0.047, β = −0.374, *p* < 0.001) and those who reported more negative emotional responses (*B* = 0.075, SE = 0.029, β = 0.169, *p* = 0.010) were more likely to engage in violent expressive behavior.

**TABLE 10:** Hierarchical multiple regression predicting tendency of violent expressive behavior for female prisoners.

Predictors	Model 1		Model 2	
	*B* (SE)	β	*B* (SE)	β
Constant	0.739*** (0.108)		1.319*** (0.286)	
Hair length (long vs. short hair)	−0.261*** (0.065)	–0.220	0.017 (0.072)	0.014
Frequency of instances of indiscipline	−0.101* (0.046)	–0.121	−0.142** (0.042)	–0.170
No. of violence-related crimes committed	−0.233 (0.578)	–0.022	−0.703 (0.525)	–0.066
Sentence length	0.000 (0.000)	0.031	0.000 (0.000)	0.033
Aggression (reactive and proactive)	0.280 (0.182)	0.085	0.127 (0.164)	0.039
Self-esteem			0.124 (0.075)	–0.096
Procedural fairness			−0.326*** (0.047)	–0.374
Negative emotional responses			0.075* (0.029)	0.169
*R* ^2^	0.074		0.259	
Adjust *R*^2^	0.059		0.240	
*R*^2^ change	0.074		0.186	
*F* for change in *R*^2^	5.034***		26.249***	

B refers to the unstandardized coefficient, β refers to the standardized coefficient, and SE refers to standard error. ***p < 0.001, **p < 0.01, *p < 0.05.

### Mediation Analysis Using Structural Equation Modeling

To further explore the pathways through which hair length would influence prisoners’ behavior, we conducted mediation analysis in which three variables—“self-esteem,” “negative emotional responses,” and “procedural fairness”—were treated as the main paths transmitting the effect of hair length to violent expressive behavior. Two other variables—“frequency of instances of indiscipline” and “aggression”—were not used as mediators because it is obvious that more instances of indiscipline and more aggression in prisons would lead to violence.

The mediation analysis was conducted using structural equation modeling (SEM) with the tool AMOS, rather than the PROCESS in SPSS. With the help of SEM, even subtle evidence of hair length exerting an effect on prisoners’ violent expression and the differences between male and female prisoners would be captured. The hypotheses for the mediation analyses are that long hair for male prisoners poses security risks by undermining self-esteem (H1a), triggering negative emotional response (H2a), and producing a sense of procedural unfairness (H3a). Similarly, short hair for female prisoners poses security risks by undermining self-esteem (H1b), triggering negative emotional responses (H2b), and producing a sense of procedural unfairness (H3b).

First, we conducted confirmatory factor analysis for scales of self-esteem, negative emotional response, and violent expressive behavior. Some items were deleted, as the factor loading did not reach the threshold of 0.5 (i.e., S2, S4, and S6 in self-esteem). In addition, some residuals were correlated for a better or acceptable model fit (i.e., e5 and e7, e8 and e9 in self-esteem; n1 and n2, n1 and n6 in negative emotional response; p1 and p2 in procedural fairness; v1 and v2 in violent expressive behavior). Three scales were adjusted to be more suitable for the mediation analysis with SEM regarding good factor loading and acceptable model fit (see Supplementary Appendix).

**FIGURE 2:**
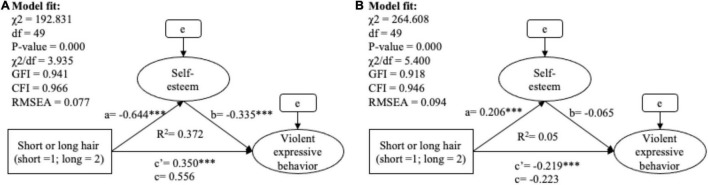
The SEM model of mediation analysis between hair length, self-esteem and violent expressive behavior among male prisoners **(A)** and female prisoners **(B)**. ***p < 0.001, **p < 0.01, *p < 0.05.

Hypothesis 1, which assumes self-esteem be a mediator to transmit the impact of hair length on violent expressive behavior, was partially supported (see Table 11 and Figure 2). Long hair lowered the self-esteem of male prisoners and further led to increased self-perceived tendencies to engage in violent expressive behavior, with the mediation effect accounting for 38% of the total effect (*B*_(indirect)_ = 0.216, SE = 0.044, β_(indirect)_ = 0.198, bootstrapping 95% CI = [0.141, 0.311]). In contrast, for female prisoners, self-esteem was not a valid mediator to transmit the effect of unwanted hair length (i.e., short hair) to violent expression (*B*_(indirect)_ = −0.013, SE = 0.013, β_(indirect)_ = −0.012, bootstrapping 95% CI = [−0.046, 0.006]). Without the mediation of self-esteem, hair length still exerted a negative impact on violent expression in the female group. Hence, Hypothesis 1a was supported while Hypothesis 1b was not.

**TABLE 11:** The mediation analysis of hair length, self-esteem and violent expressive behavior.

	*B* (SE)	*Z*	β	BootLLCI	BootULCI	*R* ^2^	Type of mediation
**Group: Male prisoners**							Partial mediation (Indirect/Total = 38%) (H1a supported)
Total effect (c)	0.566 (0.050)		0.518	0.471	0.671	0.372	
Direct effect (c′)	0.350*** (0.051)	6.915	0.321	0.240	0.472		
Indirect effect (ab)	0.216 (0.044)		0.198	0.141	0.311		
Effect of IV on mediator (a)	−0.644*** (0.055)	–11.637	–0.524			0.275	
Effect of mediator on DV (b)	−0.335*** (0.045)	–7.382	–0.377				
**Group: Female prisoners**							No mediation (H1b not supported)
Total effect (c)	−0.233 (0.052)		–0.213	–0.345	–0.134	0.050	
Direct effect (c′)	−0.219*** (0.05)	–4.350	–0.200	–0.327	–0.122		
Indirect effect (ab)	−0.013 (0.013)		–0.012	–0.046	0.006		
Effect of IV on mediator (a)	0.206*** (0.059)	3.478	0.165			0.027	
Effect of mediator on DV (b)	−0.065 (0.043)	–0.513	–0.074				

***p < 0.001, **p < 0.01, *p < 0.05.

**FIGURE 3:**
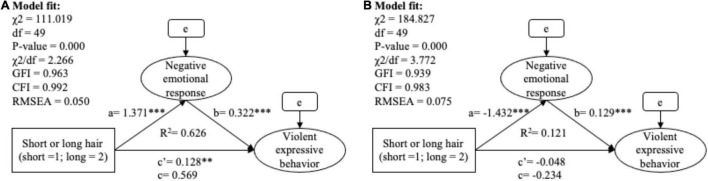
The SEM model of mediation analysis between hair length, negative emotional response and violent expressive behavior among male prisoners **(A)** and female prisoners **(B)**. ***p < 0.001, **p < 0.01, *p < 0.05.

Hypothesis 2 (a and b), which predicts that negative emotional response is a mediator between hair length and violent behavior, was fully supported (see Table 12 and Figure 3). Retaining longer hair triggered a negative emotional response of male prisoners, consequently resulting in an increase in perceived violent expressive behavior (*B*_(indirect)_ = 0.441, SE = 0.048, β_(indirect)_ = 0.402, bootstrapping 95% CI = [0.356, 0.574]), with 82% of the effect of the hair length on violent expressive behavior explained by this mediation path within the male group. The situation was quite similar in female prisoners, while a slight difference was that negative emotional response fully mediated the effect of undesired hair length on violent expression (*B*_(indirect)_ = −0.185, SE = 0.039, β_(indirect)_ = −0.169, bootstrapping 95% CI = [−0.266, −0.113]).

**TABLE 12:** The mediation analysis of hair length, negative emotional response and violent expressive behavior.

Group: Male	*B* (SE)	*Z*	β	BootLLCI	BootULCI	*R* ^2^	Type of mediation
**Group: Male prisoners**							Partial mediation (Indirect/Total = 82%) (H2a supported)
Total effect (c)	0.569 (0.05)		0.518	0.475	0.674	0.626	
Direct effect (c′)	0.128** (0.04)	3.202	0.116	0.04	0.221		
Indirect effect (ab)	0.441 (0.048)		0.402	0.356	0.574		
Effect of IV on mediator (a)	1.371*** (0.097)	14.129	0.558			0.311	
Effect of mediator on DV (b)	0.322*** (0.021)	15.436	0.72				
**Group: Female prisoners**							Full mediation (H2b supported)
Total effect (c)	−0.234 (0.052)		–0.213	–0.346	–0.136	0.121	
Direct effect (c′)	−0.048 (0.056)	–0.861	–0.044	–0.179	0.076		
Indirect effect (ab)	−0.185 (0.039)		–0.169	–0.266	–0.113		
Effect of IV on mediator (a)	−1.432*** (0.107)	–13.385	–0.525			0.275	
Effect of mediator on DV (b)	0.129*** (0.021)	6.120	0.322				

***p < 0.001, **p < 0.01, *p < 0.05.

**FIGURE 4:**
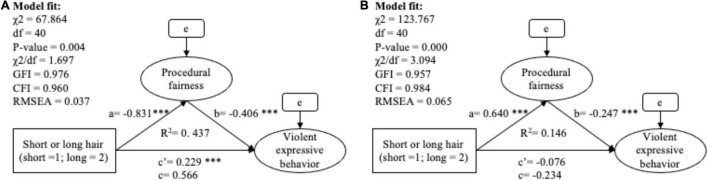
The SEM model of mediation analysis between hair length, procedural fairness and violent expressive behavior among male prisoners **(A)** and female prisoners **(B)**. ***p < 0.001, **p < 0.01, *p < 0.05.

Hypothesis 3 (a and b), which predicts procedural fairness as a mediator bridging the effect of undesired hair length on violent expression (see Table 13 and Figure 4), was fully supported by the outcome of a partial mediation in the male group (*B*_(indirect)_ = 0.377, SE = 0.042, β_(indirect)_ = 0.309, bootstrapping 95% CI = [0.261, 0.431]) and a full mediation in the female group (*B*_(indirect)_ = −0.158, SE = 0.030, β_(indirect)_ = −0.144, bootstrapping 95% CI = [−0.225, −0.105]).

**TABLE 13:** The mediation analysis of hair length, procedural fairness and violent expressive behavior.

Group: Male	*B* (SE)	*Z*	β	BootLLCI	BootULCI	*R* ^2^	Type of mediation
**Group: Male prisoners**							Partial mediation (Indirect/Total = 40%) (H3a supported)
Total effect (c)	0.566 (0.050)		0.518	0.472	0.672	0.437	
Direct effect (c′)	0.229*** (0.050)	4.542	0.209	0.133	0.345		
Indirect effect (ab)	0.337 (0.042)		0.309	0.261	0.431		
Effect of IV on mediator (a)	−0.831*** (0.056)	–14.827	–0.601			0.361	
Effect of mediator on DV (b)	−0.406*** (0.041)	–9.877	–0.514				
**Group: Female prisoners**							Full mediation (H3b supported)
Total effect (c)	−0.234 (0.052)		–0.213	–0.345	–0.136	0.146	
Direct effect (c′)	−0.076 (0.052)	–1.455	–0.069	–0.182	0.019		
Indirect effect (ab)	−0.158 (0.030)		–0.144	–0.225	–0.105		
Effect of IV on mediator (a)	0.640*** (0.067)	9.618	0.414			0.171	
Effect of mediator on DV (b)	−0.247*** (0.036)	–6.881	–0.348				

***p < 0.001, **p < 0.01, *p < 0.05.

## Conclusion

The present study found that the potential risks of individual violence are high in male prisons. Aggression scores show that male prisoners had a potential risk of engaging in aggression, scoring 15.67 out of a total of 42 (see Table 6). Consequently, it is likely that they would sometimes engage in aggression. It is observed that conflicts, fighting, revenge seeking, and gang fights by male prisoners were common (55.8% to 71.2%). The findings are in line with previous research showing that male prisoners commit more violence, and more serious violence, than their female counterparts (Craddock, 1996; Wolff et al., 2007; Sorensen and Cunningham, 2010; Wulf-Ludden, 2013). Cups, tumblers, and pens were seen to be the most common types of weapon used in the conflicts. Since the perceived risks of weapon use by prisoners are high, the chances of the occurrence of violence inside correctional institutions should not be underestimated. Correctional institutions should implement relevant security measures, including hair regulation measures, that are effective in keeping all the potential risk factors under control. The present study confirmed that one risk factor that can incite violence is the hair length requirement, in spite of gender differences. The study showed that male prisoners prefer short hair and female prisoners prefer long hair, with the possibility of violence under the contrary conditions.

For men’s prisons, the perceived risks of security threats and undisciplined behaviors increase if male prisoners retain long hair. There is a high possibility that allowing male prisoners to retain long hair would result in the hiding of weapons (44.6%) and becoming an attack target (36.6%). Long hair in male prisoners could be used to hide small and sharpened objects or self-made needle-like weapons for the purposes of self-defense, attacking other inmates, or committing suicide. This is in line with previous research showing that long hair is used in fighting or attacking tactics among male prisoners (Singh, 1997; Tjaden and Thoennes, 1998; Rippon, 2000). In addition, the present study revealed that long hair length for male prisoners would likely increase their negative emotional responses and sense of procedural unfairness, thereby leading to violent expressive behavior. It also revealed that long hair length for male prisoners would likely lower their self-esteem. This is understandable given that male prisoners’ short hair is usually regarded as a symbol of masculinity and long hair femininity, and previous studies have also identified a masculinity culture in men’s prisons in which masculine men often are positioned at the higher level of the power hierarchy when compared with feminine men (Donaldson, 2001; Hensley et al., 2003; Michalski, 2017). Requiring all male prisoners to retain short hair would be conducive to the emotional stability of male prisoners and minimize the potential danger they pose to both themselves and others.

For women’s prisons, the risk of security threats and undisciplined behaviors increases if female prisoners are required to cut their hair short. The study found that women generally have a lower risk of aggression when compared with male prisoners, as their aggression scores are very low (3.31 out of a total of 42). Conflicts and the use of weapons among female prisoners were also not common. However, if these prisoners were forced to cut their hair short, a substantial number of them would certainly engage in violent expressive behavior, such as self-harm, throwing things, hitting others, pulling others’ hair, and swearing at others (ranging from 11.8% to 20.1%), as many of them believed that nice hairstyles and length would make them feel respected and self-confident (67.1% and 72.8%, respectively). Furthermore, short hair length requirements for female prisoners would be likely to lower their perceived sense of procedural fairness of the correctional institutions and increase their negative emotional responses. Allowing female prisoners to retain long hair would thus be conducive to their emotional stability and minimize their risk of violent expressive behavior. Involuntary haircutting of female prisoners would arouse unnecessary emotional disturbance that may lead to aggression, thus causing a security risk.

To echo previous research on gender identity and hairstyles (Mesko and Bereczkei, 2004; Stenn, 2016), the present study revealed that while hairstyles may relate to the identity of a person, there are gender differences in their preferred hairstyles and identity. As a cultural and universal norm, women have longer hair than men, and they use extra resources to keep long hair looking pretty, which also becomes a status symbol (Stenn, 2016). Long hair represents beauty, femininity, physical health, and attractiveness, even in Chinese societies (Mesko and Bereczkei, 2004; Zheng, 2016) and among Chinese female prisoners. Undoubtedly, depriving them of the right to have long hair would result in emotional instability and violent expressive behavior. On the other hand, while long hair in men may symbolize anti-authority and sexuality in a society (Leach, 1958; Hallpike, 1969; Larsen and White, 1974), short hair is culturally associated with the prowess and toughness of men (Manning, 2010), or it is essential in maintaining the identities of gang members (Melde and Esbensen, 2013), especially in prisons where a culture of masculinity is dominant (Michalski, 2017). This finding is in line with the argument of Synnott (1987, p. 382) that “opposite sexes have opposite hair.”

To conclude, the present study applies a perspective of gender differences to explore concerns around restrictive hair regulations in Chinese prisons. While it found that male prisoners are inherently more tensive than the female group in terms of violent proclivities, the influence of hairstyles on behavioral responses in male and female prisoners should not be underestimated. The findings suggest that there are significant differences in the cultural meanings of hairstyles between men and women, which would affect the management of prisons. Violent behavior is associated with hairstyles, and the influence path is gender related. Long hair in male prisons would lead to security and violence risks, but this is not the case in female prisons. Hairstyles that do not meet social norms would decrease male prisoners’ self-esteem, while increasing all prisoners’ negative emotional responses and reducing their perceived procedural fairness.

The present study examined a rare research topic: hairstyles and gender differences in violence in Chinese prisons. Given that many prisons in Asian and African nations have an authoritarian style of governance similar to that of China, this study is of considerable international relevance. It concludes that hair regulation is needed to ensure the day-to-day operations of correctional institutions for two reasons: the maintenance of security, and the maintenance of prisoners’ mental well-being. From a prison management perspective, hair regulation is an essential policy in correctional institutions for maintaining workplace safety, hygiene, security, and discipline. However, the implementation of any hair-regulating policy should consider gender needs and differences, or else it would induce prisoners’ negative emotions and violent expressive behavior. Our findings and conclusions do not concur with the aforementioned court judgment on safeguarding prisoners’ equality and preventing sex discrimination. Any hair-regulation policy should respect unique social and cultural meanings and gender differences and address the negative impact on prisoners’ emotion and self-identity. In particular, forcing female prisoners to cut their hair short harms not only their body (Holton, 2020) and femininity (Manning, 2010) but also their self (Fabry, 2016), thus shaming them by taking away their sexual identity (Warring, 2006). This act in itself serves as a secondary punishment and constitutes gender-based violence (Labotka, 2014; Ruberg, 2019). Based on the hair length impacts on prisoners, we recommend short hair length for male prisoners and long hair length for female prisoners in Chinese prisons if a hair-regulation policy has to be implemented.

## Data Availability Statement

The original contributions presented in the study are included in the article/Supplementary Material, further inquiries can be directed to the corresponding author.

## Ethics Statement

The studies involving human participants were reviewed and approved by the Correctional Services Department of Hong Kong. The patients/participants provided their written informed consent to participate in this study.

## Author Contributions

TWL organized, reviewed, and edited the manuscript. CH organized the database and wrote the first draft of the manuscript. XG contributed to data analysis. SK contributed to literature review. All authors contributed to manuscript revision, read, and approved the submitted version.

## Conflict of Interest

The authors declare that the research was conducted in the absence of any commercial or financial relationships that could be construed as a potential conflict of interest.

## Publisher’s Note

All claims expressed in this article are solely those of the authors and do not necessarily represent those of their affiliated organizations, or those of the publisher, the editors and the reviewers. Any product that may be evaluated in this article, or claim that may be made by its manufacturer, is not guaranteed or endorsed by the publisher.
