# Supplementary material for: Prisoners’ Perceived Violence and Hair Regulation in Hong Kong Prisons: Gender-Based Differences
Source: Front Psychol. 2022 Apr 27;13:869898. doi: 10.3389/fpsyg.2022.869898 (PMC9093688; doi:10.3389/fpsyg.2022.869898)
Supplement: Supplementary file 1 [file Data_Sheet_1.docx]

**Appendix 1.** Factor loadings, and model fit, Composite reliability, discriminant validity and correlation coefficients of self-esteem, negative emotional response, violent expressive behavior.

**Appendix 1a.** Factor loadings and model fit before adjusting.

| Factor and item | Factor loading | χ2 | df | P-value | χ2/df | GFI | CFI | RMSEA |
| --- | --- | --- | --- | --- | --- | --- | --- | --- |
| ***Self-esteem*** |  | 1774.612 | 27 | 0.000 | 65.726 | 0.646 | 0.649 | 0.255 |
| S1: I feel that I have a number of good qualities | 0.749 |  |  |  |  |  |  |  |
| S2: I take a positive attitude toward myself | 0.839 |  |  |  |  |  |  |  |
| S3: I feel that I am no good at all * | 0.389 |  |  |  |  |  |  |  |
| S4: I feel that I am a person of worth, at least on an equal plane with others | 0.757 |  |  |  |  |  |  |  |
| S5: I would be inclined to feel that I am a failure * | 0.492 |  |  |  |  |  |  |  |
| S6: I feel that I am able to do things as well as most other people | 0.742 |  |  |  |  |  |  |  |
| S7: I feel that I do not have much to be proud of * | 0.473 |  |  |  |  |  |  |  |
| S8: I feel useless * | 0.543 |  |  |  |  |  |  |  |
| S9: I am satisfied with myself | 0.732 |  |  |  |  |  |  |  |
| ***Negative emotional response*** |  | 121.271 | 9 | 0.000 | 13.475 | 0.964 | 0.989 | 0.112 |
| N1: if the hair length requirement would be enforced, I would feel disrespected | 0.943 |  |  |  |  |  |  |  |
| N2: if the hair length requirement would be enforced, I would feel inferiority | 0.958 |  |  |  |  |  |  |  |
| N3: if the hair length requirement would be enforced, I would feel furiousness | 0.954 |  |  |  |  |  |  |  |
| N4: if the hair length requirement would be enforced, I would feel bitterness | 0.948 |  |  |  |  |  |  |  |
| N5: if the hair length requirement would be enforced, I would feel anxiety | 0.928 |  |  |  |  |  |  |  |
| N6: if the hair length requirement would be enforced, I would feel sadness | 0.961 |  |  |  |  |  |  |  |
| ***Procedural fairness*** |  | 77.485 | 5 | 0.000 | 15.497 | 0.969 | 0.985 | 0.120 |
| P1: CI staff treat prisoners with respect | 0.899 |  |  |  |  |  |  |  |
| P2: CI staff treat prisoners fairly | 0.911 |  |  |  |  |  |  |  |
| P3: CI staff explain their decisions to prisoners | 0.881 |  |  |  |  |  |  |  |
| P4: CI staff make decisions based on facts and not personal opinions | 0.885 |  |  |  |  |  |  |  |
| P5: CI staff take the time to listen to prisoners | 0.876 |  |  |  |  |  |  |  |
| ***Violent expressive behavior*** |  | 102.813 | 5 | 0.000 | 20.563 | 0.959 | 0.979 | 0.140 |
| V1: if hair was required to be a short/long length, I would harm myself | 0.812 |  |  |  |  |  |  |  |
| V2: if hair was required to be a short/long length, I would throw things | 0.863 |  |  |  |  |  |  |  |
| V3: if hair was required to be a short/long length, I would hit others | 0.915 |  |  |  |  |  |  |  |
| V4: if hair was required to be a short/long length, I would pull other’s hair | 0.902 |  |  |  |  |  |  |  |
| V5: if hair was required to be a short/long length, I would threaten/swear others | 0.889 |  |  |  |  |  |  |  |
| Note: The number of error term for each item was same as the item number with a lower-case letter at the front. The error term for self-esteem is s1 to s9, the error term for negative emotional response is n1 to n6, the error term for procedural fairness is p1 to p5, and the error term for violent expressive behavior is v1 to v5. | | | | | | | | |

**Appendix 1b.** Factor loadings and model fit after adjusting.

| Factor and item | Factor loading | χ2 | df | P-value | χ2/df | GFI | CFI | RMSEA |
| --- | --- | --- | --- | --- | --- | --- | --- | --- |
| ***Self-esteem*** |  | 22.007 | 7 | 0.003 | 3.114 | 0.993 | 0.995 | 0.046 |
| S1: I feel that I have a number of good qualities | 0.786 |  |  |  |  |  |  |  |
| S2: I take a positive attitude toward myself | 0.873 |  |  |  |  |  |  |  |
| S4: I feel that I am a person of worth, at least on an equal plane with others | 0.762 |  |  |  |  |  |  |  |
| S6: I feel that I am able to do things as well as most other people | 0.738 |  |  |  |  |  |  |  |
| S8: I feel useless * | 0.518 |  |  |  |  |  |  |  |
| S9: I am satisfied with myself | 0.713 |  |  |  |  |  |  |  |
| ***Negative emotional response*** |  | 49.482 | 7 | 0.000 | 7.069 | 0.984 | 0.996 | 0.078 |
| N1: if the hair length requirement would be enforced, I would feel disrespected | 0.932 |  |  |  |  |  |  |  |
| N2: if the hair length requirement would be enforced, I would feel inferiority | 0.954 |  |  |  |  |  |  |  |
| N3: if the hair length requirement would be enforced, I would feel furiousness | 0.956 |  |  |  |  |  |  |  |
| N4: if the hair length requirement would be enforced, I would feel bitterness | 0.950 |  |  |  |  |  |  |  |
| N5: if the hair length requirement would be enforced, I would feel anxiety | 0.924 |  |  |  |  |  |  |  |
| N6: if the hair length requirement would be enforced, I would feel sadness | 0.964 |  |  |  |  |  |  |  |
| ***Procedural fairness*** |  | 16.996 | 4 | 0.002 | 4.429 | 0.993 | 0.997 | 0.057 |
| P1: CI staff treat prisoners with respect | 0.874 |  |  |  |  |  |  |  |
| P2: CI staff treat prisoners fairly | 0.888 |  |  |  |  |  |  |  |
| P3: CI staff explain their decisions to prisoners | 0.888 |  |  |  |  |  |  |  |
| P4: CI staff make decisions based on facts and not personal opinions | 0.896 |  |  |  |  |  |  |  |
| P5: CI staff take the time to listen to prisoners | 0.885 |  |  |  |  |  |  |  |
| ***Violent expressive behavior*** |  | 17.722 | 4 | 0.001 | 4.431 | 0.993 | 0.997 | 0.059 |
| V1: if hair was required to be a short/long length, I would harm myself | 0.788 |  |  |  |  |  |  |  |
| V2: if hair was required to be a short/long length, I would throw things | 0.845 |  |  |  |  |  |  |  |
| V3: if hair was required to be a short/long length, I would hit others | 0.922 |  |  |  |  |  |  |  |
| V4: if hair was required to be a short/long length, I would pull other’s hair | 0.907 |  |  |  |  |  |  |  |
| V5: if hair was required to be a short/long length, I would threaten/swear others | 0.890 |  |  |  |  |  |  |  |
| Note: The number of error term for each item was same as the item number with a lower-case letter at the front. The error term for self-esteem is s1 to s9, the error term for negative emotional response is n1 to n6, the error term for procedural fairness is p1 to p5, and the error term for violent expressive behavior is v1 to v5. | | | | | | | | |
